# The non-cylindrical crustal architecture of the Pyrenees

**DOI:** 10.1038/s41598-018-27889-x

**Published:** 2018-06-25

**Authors:** Sébastien Chevrot, Matthieu Sylvander, Jordi Diaz, Roland Martin, Frédéric Mouthereau, Gianreto Manatschal, Emmanuel Masini, Sylvain Calassou, Frank Grimaud, Hélène Pauchet, Mario Ruiz

**Affiliations:** 1 GET, Université de Toulouse, CNRS, IRD, UPS, Toulouse, France; 2 IRAP, Université de Toulouse, CNRS, CNES, UPS, Toulouse, France; 3 Institute of Earth Sciences Jaume Almeria, ICTJA-CSIC, Barcelona, Spain; 4 IPGS/EOST, Université de Strasbourg, Strasbourg, France; 5 R&D, CSTJF - Total S.A., Pau, France

## Abstract

We exploit the data from five seismic transects deployed across the Pyrenees to characterize the deep architecture of this collisional orogen. We map the main seismic interfaces beneath each transect by depth migration of P-to-S converted phases. The migrated sections, combined with the results of recent tomographic studies and with maps of Bouguer and isostatic anomalies, provide a coherent crustal-scale picture of the belt. In the Western Pyrenees, beneath the North Pyrenean Zone, a continuous band of high density/velocity material is found at a very shallow level (~10 km) beneath the Mauleon basin and near Saint-Gaudens. In the Western Pyrenees, we also find evidence for northward continental subduction of Iberian crust, down to 50–70 km depth. In the Eastern Pyrenees, these main structural features are not observed. The boundary between these two domains is near longitude 1.3 °E, where geological field studies document a major change in the structure of the Cretaceous rift system, and possibly a shift of its polarity, suggesting that the deep orogenic architecture of the Pyrenees is largely controlled by structural inheritance.

## Introduction

**Figure 1:**
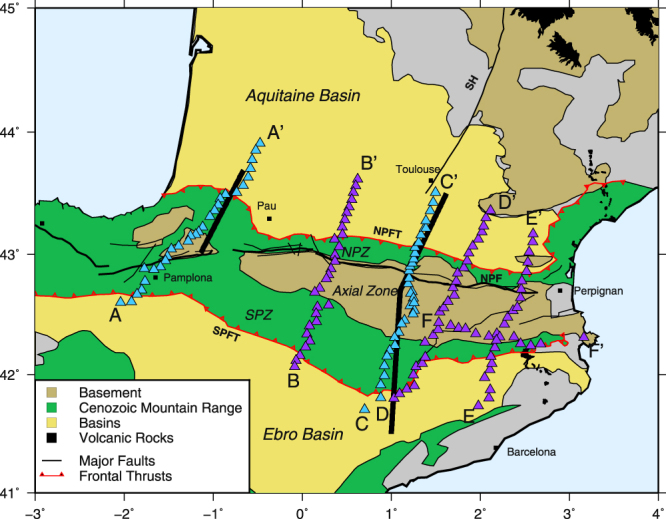
Main geological and structural units of the study region. NPF: North Pyrenean Fault, NPFT: North Pyrenean Front Thrust, SPFT: South Pyrenean Front Thrust, SH: Sillon Houiller (sometimes also referred to as the “Toulouse Fault”), NPZ: North Pyrenean Zone, SPZ: South Pyrenean Zone. The triangles show the stations of the PYROPE (blue) and OROGEN transects (purple). The thick black lines show the position of the ECORS-Pyrenees and ECORS-Arzacq deep seismic sounding profiles, respectively located in the Central and Western Pyrenees.

The Pyrenees are a collisional orogen produced by the convergence between the Iberian and Eurasian plates from late Cretaceous (~85 Ma) to early Miocene (~20 Ma). The mountain belt is made of a stack of Iberian and European crustal units forming a doubly vergent orogenic wedge. These units are the Axial Zone (AZ), mainly formed by Hercynian basement rocks, the North Pyrenean Zone (NPZ), consisting mainly in thick Mesozoic sediments, and the fold-and-thrust belt of the South Pyrenean Zone (SPZ) (Fig. 1). The limit between the AZ and the NPZ corresponds to the North Pyrenean Fault (NPF), which is classically interpreted as the former plate boundary between the Iberian and European plates^1^. The Pyrenees are flanked by two flexural foreland basins, the Aquitaine (AB) and Ebro (EB) basins, which are respectively overthrust by the NPZ and the SPZ.

**Figure 2:**
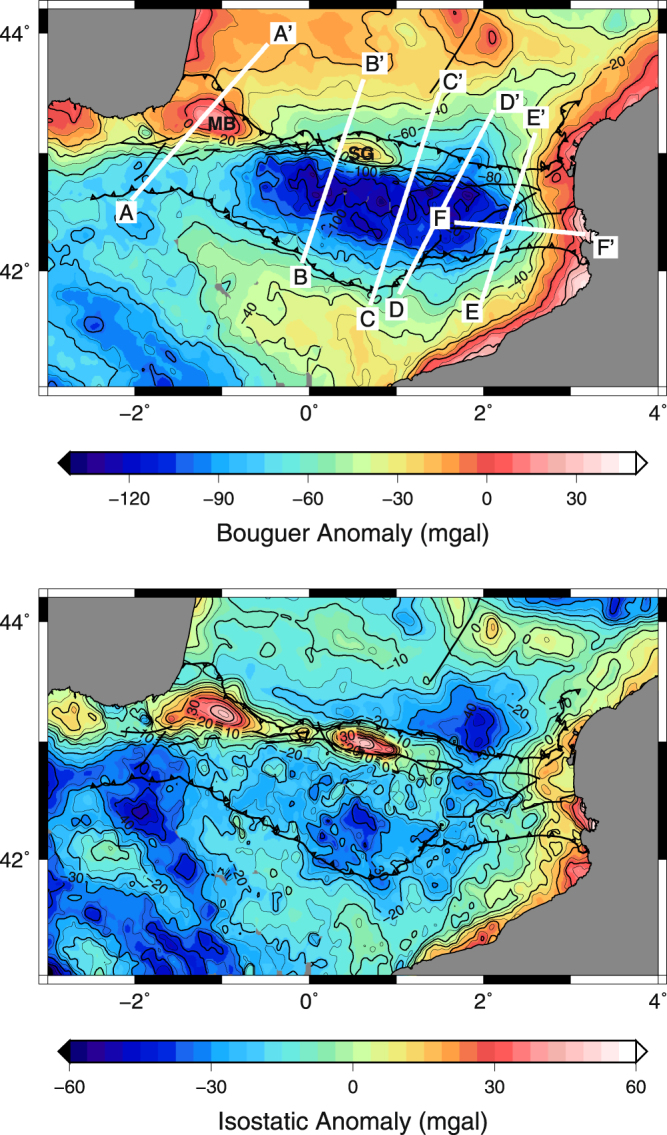
Map of computed Bouguer (top) and isostatic (bottom) gravity anomalies. MB: Mauleon Basin anomaly and SG: Saint-Gaudens anomaly.

A first indication of the asymmetrical structure of the Pyrenean roots is expressed in the map of Bouguer gravity anomalies (Fig. 2), computed from the Pyrenean gravity data distributed by the Bureau Gravimétrique International (BGI)^2^. Strong positive anomalies are only observed on the northern flank of the mountain belt, in the NPZ, with prominent anomalies beneath the Mauleon basin (MB), and Saint-Gaudens (SG). The origin of these anomalies has been the source of long-lasting controversies and was attributed either to slices of mantle or lower crustal material emplaced at a shallow crustal level^3–5^. A recent tomographic study relying on teleseismic P waves recorded by the Western PYROPE transect (transect A-A’ in Fig. 1) showed that the Mauleon Basin anomaly is produced by a mantle body whose top lies at about 10 km depth^6^. Mantle exhumation has been interpreted to occur during the Aptian episode of rifting^7–10^, suggesting that the band of positive Bouguer anomalies represents the former hyper-extended distal domain of the European-Iberian rift.

The ECORS-Pyrenees profile (Fig. 1) provided further evidence for the N-S asymmetry of the deep architecture of the Pyrenees with the northward underthrusting of the Iberian plate beneath the European plate^11,12^. It also showed that the mountain root is essentially made of thickened Iberian crust, in good agreement with the results of earlier long-range deep reflection studies^13,14^. The ECORS-Arzacq profile (Fig. 1) was shot a few years later in the Western Pyrenees. While no deep reflectors could be clearly identified in this section, interpretations also favored the northward subduction of the Iberian plate^15,16^. This was recently confirmed by migration of receiver functions from two dense transects (transects A-A’ and C-C’ in Fig. 1) deployed during the PYROPE project^17^ and by full waveform inversion tomography^6^. Up to now, reconstructions of the Pyrenees mainly relied on these two sections in the Central and Western Pyrenees^11,16,18–20^, and were often extrapolated to the whole mountain range. Here, we exploit the data from six transects, which were deployed during the PYROPE and OROGEN experiments (Fig. 1) to map the main seismic discontinuities by depth migration of P-to-S conversions. These six transects provide a unique dataset to investigate the lateral variations of the deep architecture of a mountain belt.

## Data and Method

### The PYROPE and OROGEN transects

During the PYROPE project^21,22^, in addition to the backbone array of 50 temporary broad-band sensors, two additional dense transects equipped with medium-band GURALP CMG40 sensors were deployed across the Central and Western Pyrenees (transects A-A’ and C-C’ shown with blue triangles on Fig. 1). These transects approximately followed the ECORS-Pyrenees and ECORS-Arzacq profiles. The first transect had 37 stations recording from October 2011 to October 2012. The second transect had 29 stations recording from October 2012 to October 2013. The initial motivation of these deployments was to investigate the advantages and shortcomings of active and passive imaging studies through a detailed comparison between ECORS seismic reflection sections and migrated receiver function sections. The results of this comparison were reported in^17^. The quality of the receiver function (RF) sections stimulated the deployment of three new transects (violet triangles in Fig. 1), in order to characterize the lateral variations of crustal structures, in particular in the Eastern Pyrenees, which remained until today poorly constrained. These transects were funded by the OROGEN project, a tripartite research program that involves the ‘Centre National de la Recherche Scientifique’ (CNRS), the ‘Bureau des Recherches Géologiques et Minières’ (BRGM) and TOTAL oil company. The third transect (28 receivers), deployed from November 2013 to October 2014, was located approximately at equal distance from the two first PYROPE transects. The last two transects were deployed in the Eastern Pyrenees. The goal of the easternmost transect (E-E’ in Fig. 1), deployed from April 2015 to May 2016, was to investigate the eastern termination of the Pyrenees, affected by the Neogene extension related to the retreat of the Tyrrhenian slab. For this purpose, to better document the transition from the thickened Pyrenean domain to the thinned post-orogenic Gulf of Lion rift system, we deployed an additional orthogonal transect on the southern side of the Eastern Pyrenees (transect F-F’ in Fig. 1). The results obtained from these two perpendicular transects are discussed in a companion paper^23^. The last transect (transect D-D’ in Fig. 1) was deployed from June 2016 to February 2017 approximately at equal distance from the central and eastermost profiles. The motivation for this final transect was to sample the Eastern Pyrenean domain, presumably not affected by the opening of the Western Mediterranean basin.

### Data selection

In this study, we focus on P-to-S conversions to map the main seismic interfaces beneath the different transects. For the sake of consistency, we apply the same data analysis procedure as in^17^ on the different transects. We select the P wave records corresponding to teleseismic sources in the distance range 28°–95° with moment magnitude larger than 6. The seismograms are cut 50 s before and 250 s after the P wave onset and the horizontal components are rotated to obtain the radial and transverse components. The receiver functions are then computed by deconvolving the radial component from the vertical component, using the iterative deconvolution method by^24^ with a 2 Hz Gaussian filter. For each event, the section of receiver functions sorted according to epicentral distance is visually inspected to check that a coherent signal corresponding to the Pms phase (the P-to-S conversion on the Moho discontinuity) is present a few seconds after the P wave arrival. If this is the case, then the receiver functions corresponding to this event are kept for the migration.

### Depth migration of receiver functions by CCP stack

We apply a simple’common conversion point’ (CCP) stack approach^25,26^ to map the main seismic interfaces beneath each transect down to 100 km depth. The principle of this approach is to assume that all the radial component wavefield recorded in the coda of the P wave is produced by P-to-S conversions of the incoming teleseismic P wave at depth. This assumption is obviously not valid, because other types of waves are also present in the seismograms like for example back-scattered waves on shallow discontinuities. However, their contribution will be reduced by stacking a sufficiently large number of traces characterized by different backazimuths and incidence angles. While including multiples could potentially improve the results of migration^27^, their interest for imaging seismic interfaces beneath the Pyrenees is rather limited owing to the extremely strong complexity of the crust. Indeed, coherent multiples are almost never observed on event receiver function sections, if we exclude the multiple reflections in the shallow sediment layers of the Aquitaine and Ebro basins. For the westernmost transect, the excellent agreement between the migrated section^17^, and the shear velocity model derived from full waveform inversion^6^ suggests that CCP stack is a very efficient means to obtain robust, first-order pictures of the main seismic interfaces beneath a mountain belt.

We use the crustal velocities of the ak135 reference Earth model^28^ with a lower crust extended down to 60 km depth to backpropagate the rays downwards, starting from the position of the receivers at the surface. Raytracing is performed in 3D, inside a grid composed of 1 km cubic cells. We compute the average amplitude of all the potential converted phases that reach each cell, and apply a horizontal smoothing filter. The size of the smoothing filter is taken as the Fresnel zone width $\sqrt{\lambda z}$, with *λ* = 4 km and z the depth of the cell in km.

### Results

**Figure 3:**
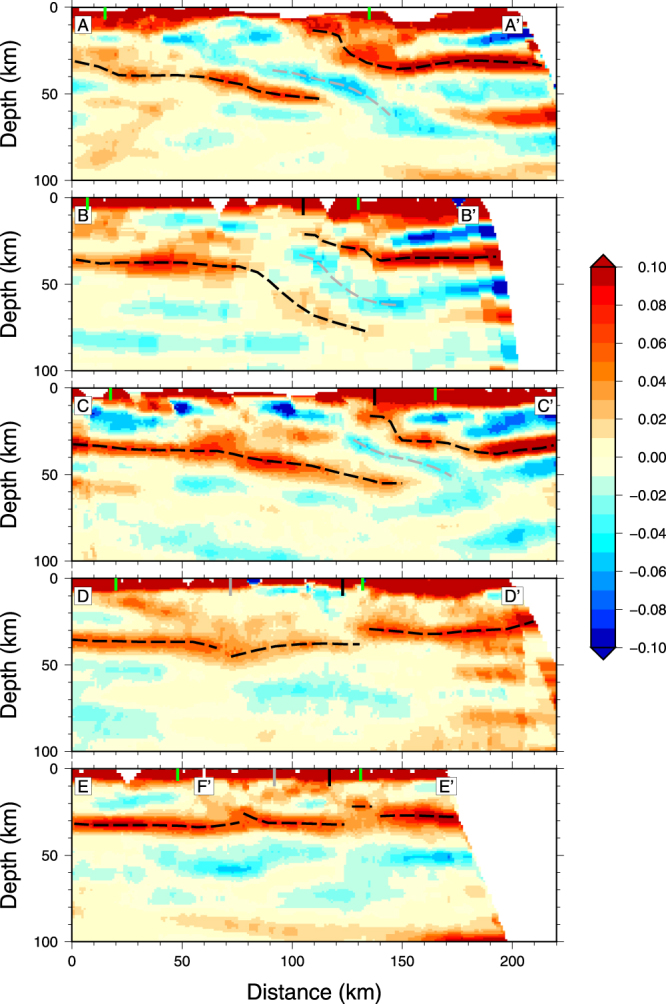
Common Conversion Point sections for (from top to bottom) transects (**A**-A’), (**B**-B’), (**C**-C’), (**D**-D’) and (**E**-E’) (see Fig. 1 for the localization of these transects). The Iberian and European Moho are represented with black dashed lines and the top of the subducting Iberian crust with a grey dashed line. The vertical lines show the positions of the SPFT and NPFT (green), the NPF (black, and of the Têt Fault (grey). The position of the crossing of transect (**E**-E’) with transect (**F**-F’) is also indicated.

Figure 3 presents the migrated sections obtained for the five transects, sorted from west (top) to east (bottom). The sign convention is such that for discontinuities characterized by positive shear velocity jumps downward (e.g. the Moho), the conversions show in red.

The westernmost profile (A-A’ in Fig. 3) clearly shows two distinct and disconnected Mohos beneath the Iberian and European plates. Beneath the SPZ, the Iberian Moho is strongly dipping to the north, with a rather small amplitude and a flat section beneath the AZ before plunging beneath the European plate. The European Moho is flat beneath the Aquitaine basin, around 30 km depth, and presents two steps at respectively 25 and 10 km depth. Note that beneath the Aquitaine basin, the section is contaminated by the strong reverberations that follow the main seismic phases (P, Pms, etc…) produced in a shallow low-velocity sedimentary layer. As already mentioned, the geometry of the Iberian and European Mohos is in excellent agreement with the tomographic model obtained by full waveform inversion of teleseismic waves^6^. The shallow European Moho in the migrated section and the strong positive velocity anomaly beneath the Mauleon basin seen in the tomographic model obtained by^6^ provide strong evidence for the presence of a shallow mantle body lying at about 10 km depth. This very anomalous structure has been interpreted as the distal fossilized European margin produced during the Cretaceous episode of rifting^9,29^. These new tomographic results suggest a very thin slice of European crust on top of the exhumed mantle, as observed in recent field geological studies^30,31^. Another salient feature in the migrated section is the presence of a negative polarity interface, which parallels the north-dipping Iberian Moho. This structure probably marks the top of the subducted Iberian crust. This section thus provides compelling evidence for the continental underthrusting of the lowermost Iberian crust beneath the European plate. It also explains why this thickened crustal domain is not expressed in the map of Bouguer gravity anomalies, because it is masked by the strong signature of the shallow mantle body. Note that the northern limit of the Iberian slab is poorly constrained, owing to the effect of crustal reverberations in the Aquitaine Basin that contaminate the waveforms.

The Saint-Gaudens profile (transect B-B’ in Fig. 3) shows a very similar general structure, with underthrust Iberian crust that can be followed down to about 70 km depth. The shallowing of the mantle beneath the NPZ is still observed but less pronounced, the European mantle lying at about 20 km depth. As can be seen in Fig. 2, this profile just passes west of the Saint-Gaudens positive Bouguer anomaly. The very sharp contours of this anomaly, even sharper than those of the Mauleon Basin anomaly in the Western Pyrenees, suggest that its limits are almost vertical and that the mantle is at a shallower level than beneath the Mauleon basin (~7–8 km). These observations point to a continuous mantle body beneath the NPZ in the Western Pyrenees. However, as can be seen in the map of isostatic anomalies (Fig. 2), its geometry and distance from the surface show strong and sharp lateral variations.

The central profile (transect C-C’ in Fig. 3) approximately follows the ECORS-Pyrenees line. It samples a domain where no prominent positive Bouguer anomaly is observed (Fig. 2). However, the underthrusting of the Iberian crust beneath the European plate and the mantle body at around 20 km depth are still observed in this section. Therefore, this section presents strong similarities with the first two sections in the Western Pyrenees. The Iberian and European Mohos also show an excellent agreement with those seen in the ECORS-Pyrenees section^11^. Compared to the sections located further west, the main differences are the smaller dip of the downgoing Iberian crust and a larger thickness of subducted material.

In the Castelnaudary profile (transect D-D’ in Fig. 3), which is located about 50 km east from the central profile, the European Moho is flat and at a constant depth of 30 km. Its detection is particularly clean, with no contamination from shallow reverberations. The Iberian Moho is also rather flat, at around 37 km depth. However, a third crustal domain is observed, squeezed between the Iberian and European domains, in which the Moho is dipping to the south. Note also the north-dipping positive discontinuity that cuts the crust beneath the SPZ. This structure may correspond to the inversion of a former major crustal-scale detachment fault. But the most salient feature in this transect is the absence of northward underthusting of the Iberian crust. Clearly, this section reveals a completely different crustal architecture of the Eastern Pyrenees, contrasting with all the geological interpretations so far.

In the easternmost profile (transect E-E’ in Fig. 3), we also observe a crustal architecture with three distinct domains. The Moho is almost flat, lying at a depth of around 32, 30 and 28 km respectively in the Iberian, central and European domains. Interestingly, a sharp upward Moho offset is observed at the limit between the central and European blocks, in excellent agreement with the results of the wide-angle reflection profiles that were shot in the early 80 s^13,32^, for which the critical Moho reflections showed a similar Moho step just north of the North Pyrenean Fault.

## Discussion

### Limitations of receiver function migration

In this study, we mainly focused on the Moho used as a structural marker to constrain the large-scale crustal architecture of the Pyrenean orogen. Other secondary crustal interfaces are also detected, but because of rather large distance between the different transects, are difficult to correlate laterally from one section to the other. Nevertheless, their detection suggests that the Pyrenean belt is organized by large-scale intra-crustal structures, which remain poorly constrained owing to the limitations of classical passive imaging approaches and to the sparse spatial coverage. In addition, receiver function migration only constrains the geometry of the main seismic interfaces and not the physical properties (such as density or seismic velocities) inside the different crustal blocks. As a result, our interpretations rely on partial and incomplete structural and compositional informations. For instance, the nature and depth extent of the underthrust materials remain largely elusive, in spite of their crucial importance for the reconstruction of the Pyrenean domain before convergence. Therefore, the tomographic images recently obtained in the Western Pyrenees by full waveform inversion of teleseismic waves^6^ open new perspectives for the study of orogens. While this technique remains computationally demanding and rather complex to implement, we expect a quick democratization of its use in the near future, thanks to the increasing power of computers and to a new generation of more efficient and flexible codes that are currently under development. The other limitation comes from the geometry of current deployments, in which seismic sensors are deployed along quasi-linear profiles. Our results clearly demonstrate that the architecture of orogenic belts is 3-D, and not 2-D, with rather sharp crustal-scale transitions. Future experiments should thus address the challenge of imaging orogenic structures with denser, 3-D acquisitions.

### Crustal extension in the Eastern Pyrenees

**Figure 4:**
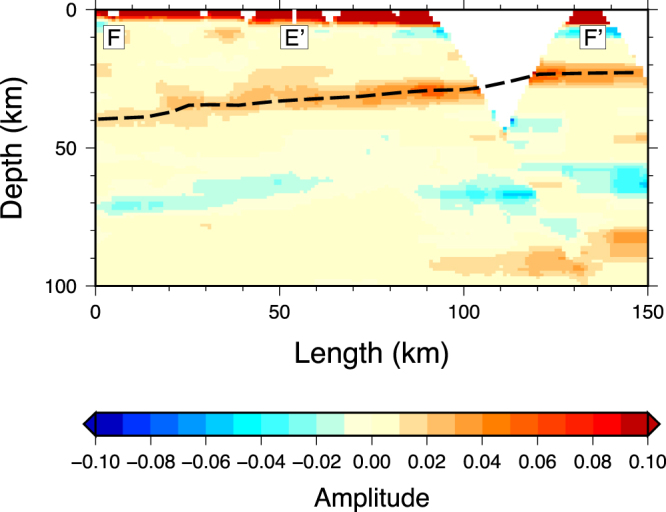
Common Conversion Point sections for transects F-F’ (see Fig. 1 for the localization of this transect). The Moho in the different crustal domains is delineated with black dashed lines. The position of the crossing with transect E-E’ is also indicated.

Earlier seismic reflection studies evidenced that the sharp contrast in Moho depth beneath the NPF observed in the Central Pyrenees is not observed in the Eastern Pyrenees^32,33^. A more recent deep seismic sounding study covering the transition from the Pyrenean range to the Mediterranean Sea confirmed the absence of crustal roots beneath the eastern termination of the Pyrenees^34^, with a Moho rising progressively from 30 km depth to ~20 km near the coast, over a distance of about 50 km. The migrated section for transect F-F’ (Fig. 4) is in remarkable agreement with the results of the earlier controlled-source experiments. The eastward reduction of crustal thickness in the Eastern Pyrenees is classically attributed to the overprint of the Oligocene rifting of the Gulf of Lion and the trench rollback of the Tyrrhenian slab^35–37^. As pointed out by^38^, the support of a 2000 m high topography in the absence of thick crustal roots requires a large buoyancy contribution of the mantle. Joint modeling of gravimetry, topography, and heat-flow data indicates a significant thinning of the mantle lithosphere^37^. A thermal erosion of the lithosphere could explain the eastward migration of extension during the last 10 Ma and the crustal buoyancy and topography uplift^39^. However, the exhumation patterns in the Eastern Pyrenees show syn-orogenic exhumation ages that are older than 30 Ma. Extension post-dating 30 Ma in the Eastern Pyrenees and simultaneous to the opening of the Gulf of Lion is only observed locally (e.g. along the Têt fault^40^) and is of minor importance. Uplift in the Eastern Pyrenees after 12 Ma^40^ correlates with large-scale exhumation at ca. 9 Ma in the Central and Eastern Pyrenees^41,42^. We infer that the process of thermal and/or mechanical erosion of the lithosphere triggered by slab retreat might have played a role but based on our data and the current geological constraints it seems unlikely that the overprint of a Oligo-Miocene extension could have erased the signature of a subduction of the Iberian plate in the Eastern Pyrenees.

### The E-W non-cylindricity of the Pyrenees

Both seismic and gravimetric data suggest that in the Western Pyrenees, a band of shallow mantle lies above fragments of subducting Iberian crust beneath the NPZ, while in the Eastern Pyrenees, these features are not observed. We have computed the map of isostatic anomalies, following the approach described in^43^. The principles of this computation is to assume that the whole region is in isostatic equilibrium. By balancing the weight of each column of rock above a compensation level, one can deduce the Moho depth from surface topography and remove the contribution from the deep crustal roots in the gravimetric signal. We considered a reference crustal column with a 30 km thick crust and a density contrast of 0.3 between crust and mantle^2^. The map of isostatic anomalies (Fig. 2) enhances the continuity of positive gravity anomalies in the NPZ, which can be followed as far east as 1.3 °E of longitude, i.e. a few tens of kilometers east of the central ECORS-Pyrenees transect, where the gravity anomaly ends abruptly. The location of the transition between the Western and Eastern Pyrenean domains is also in excellent agreement with a recent 3D model of the Pyrenees built from geological and gravity data^44^.

The apparent absence of a shallow mantle in the Eastern Pyrenees (east of 1.3 °E) could suggest that rifting did not affect the eastern domain^5^. However, this is unlikely because the stratigraphic records in the NPZ and Aquitaine basins document the tectonic thinning of the European crust during the Albian^7,29,45,46^. Moreover, the thermal imprint of rifting is present along the whole range and clearly shows that the eastern part of the Pyrenees was also affected by rifting^46–48^.

A possible explanation would be to invoke a major change in rift architecture between the Western and Eastern Pyrenees, as recently proposed in several studies. Detailed geological field studies^9^ argue for a polarity flip of the rift system around the longitude of the Toulouse Fault while the analysis of the flexural response in foreland basins^49^ suggests that the northern limit of Cretaceous rifting stepped south east of the Toulouse Fault. The Pyrenees also exhibit significant along-strike variations of the style of deformation^50^, which have been attributed to lateral variations of the rift structure, with the closure of one large basin in the Western Pyrenees^8^ and distributed extension over a wide area resulting in the formation of several basins in the Eastern Pyrenees^45,46,51^. These differences were attributed to inherited Variscan crustal composition with a more felsic and thus weaker crust in the Eastern Pyrenees, resulting in more distributed extensional deformation^50^.

While these new results regarding the architecture of the Pyrenees clearly ask for further geological and geophysical studies, we will now propose a tentative interpretation of our observations. In rifted margins, extensional processes lead to the removal of the continental crust, leading to its embrittlement, and eventually creating faults that cut from the crust into the mantle. These faults will permit the water to penetrate and serpentinize the mantle, and finally to exhume the subcontinental mantle at the seafloor. Since serpentinization considerably reduces the strength of the mantle^52^, this can lead to the development of a zone of weakness that can be later reactivated during the compressive stage^53^. In the Western Pyrenees, the rifting was localized in a single wide basin (the Mauleon Basin) in contrast to the Eastern Pyrenees where the weaker crust experienced a more distributed deformation. It is thus plausible that a much larger domain of exhumed serpentinized mantle developed in the Western Pyrenees, promoting strain localization and initiation of subduction. In contrast, in the Eastern Pyrenees, the rift basins were too narrow for these processes to occur, and compressive deformation was distributed over a much broader domain. At the initiation of convergence, topography was thus produced first in the Eastern Pyrenees, while the wide hyper-extended domain was reactivated but remained below sea level in the Western Pyrenees^54^. This explains why topography formed earlier in the Eastern Pyrenees and why crustal restorations tend to show larger amounts of shortening toward the east.

## Conclusions

The exploitation of recent dense transects deployed across the Pyrenees provide new insights into the general architecture of the Pyrenees. Underthrusting of the Iberian crust is only observed in the Western and Central Pyrenees, in a domain where a continuous band of exhumed mantle is present in the North Pyrenean Zone. The occurence of exhumed mantle before the onset of convergence seems to suggest a causal relationship between initiation of continental subduction and hyper-extension of the crust. We propose that the causal effect is related to the mechanical weakening of the mantle resulting from serpentinization along major rift structures, and perhaps also to inherited crustal weakening due the nature of the variscan basement and late Permian events. Mantle weakening likely controlled the initiation and vergence of subduction, while the effect of crustal weakening was expressed in a more distributed shortening. In any case, the Pyrenees seem to provide a convincing case for the possible occurrence of continental subduction driven by pre-orogenic inheritances rather than oceanic subduction.
